# Reliability of Ocular Aberration Measurements in Children with Moderate and Low Myopia under Scotopic Conditions

**DOI:** 10.1155/2018/2043718

**Published:** 2018-02-11

**Authors:** Zhouyue Li, Dongmei Cui, Sichun Ao, Weizhong Lan, Xiao Yang

**Affiliations:** ^1^State Key Laboratory of Ophthalmology, Zhongshan Ophthalmic Center, Sun Yat-sen University, Guangzhou, China; ^2^Aier School of Ophthalmology, Central South University, Changsha, Hunan Province, China

## Abstract

**Purpose:**

To investigate the reliability of ocular aberration measurement in myopic children under scotopic conditions and to validate the mathematical Zernike pupil scaling-down technique.

**Methods:**

Ocular aberrations of 45 myopic children were examined under scotopic conditions via iTrace aberrometer. The intra- and intersession repeatability was evaluated for both the measured values with the true pupil sizes and the estimated ones that were determined by scaling down the pupil sizes to the largest integer value across all measurements.

**Results:**

The intra- and intersession difference of clinically measured aberration was generally insignificant, and the ICCs for each aberration component exhibited good to excellent reliability (ICCs > 0.4). Similar results were found for the estimated aberration using the scaling-down technique. Although the majority of the estimated Zernike components were comparable with the corresponding measured one, the estimated values of defocus, coma, and the corresponding total aberrations were found significantly smaller than the measured values (all *P* < 0.01).

**Conclusions:**

The ocular aberration measurements in myopic children under the circumstances described are reliable. The scaling-down technique is a useful option for comparing the results obtained from different pupil sizes, but the estimated Zernike coefficients were not always comparable with the corresponding measured values.

## 1. Introduction

Myopia, which is a common cause of distance vision impairment, is reaching epidemic proportions in some Asian countries [1, 2]. Even in the United States and Europe, a dramatic increase of myopic population has been observed over the last half a century ago [3]. Myopia was estimated to affect approximately 20% of the world's population in the year 2000, and this number was predicted to increase significantly to approximately 50% in 2050 [4]. Despite the urgent situation, the exact mechanism of myopia is still not very clear. Recently, a growing body of literature has suggested that ocular aberration might play a role in myopia pathogenesis [5–7] and impact the outcome of interventions [8, 9]. It is well known that ocular aberration is very dynamic and tends to be influenced by a series of factors, such as pupil size [10, 11] and accommodation status [12–14]. These influencing factors impose more challenges for aberration measurements in children than in adults because children usually have greater pupil sizes and a more sensitive accommodation tonus. In addition, the changing course of myopia extends throughout the adolescent period, which requires multiple follow-up visits. The reliability of related measurement, including ocular aberration, is therefore very critical for correctly evaluating the disorder as well as the performance of any interventions.

Although the application of cycloplegic agents could stabilize the pupil size and accommodation status, this manner is not welcomed in clinical practice due to the side effects of the cycloplegic agents (e.g., transient photophobia and blurred near vision). An alternative method might be applied to measure the ocular aberration in a scotopic condition. However, the repeatability of the results under such circumstances in children is not well documented. Therefore, the purpose of the study was to assess the reliability of this method in children with myopia. In addition, many instruments offer an estimation of aberration that uses the pupil scaling-down technique [15] to facilitate the comparison of results obtained from different pupil sizes. The second purpose of the study was to evaluate the validity of this “simulation” technique in this population.

## 2. Subjects and Methods

### 2.1. Subjects

A total of 45 myopic children were recruited into the study, with ages ranging from 8 to 15 years (11.2 ± 1.7 years). Prior to the study, written consent was obtained from all children and their parents after a thorough explanation of the purposes and risks of all procedures throughout the study was provided. The study was conducted in accordance with the tenets of the Declaration of Helsinki and was approved by the ethical committee of Zhongshan Ophthalmic Center, Sun Yat-sen University. Before the study, each participant underwent an ophthalmic examination to ensure ocular health. All subjects had a best spectacle-corrected visual acuity of logMAR 0.00 or better. The mean spherical equivalent refractive error (SER) was −2.72 ± 0.87 DS (range: −4.00~−0.50 DS). The mean axial length was 24.82 ± 0.76 mm (range: 23.16~26.16 mm). No subject had systemic diseases that might induce ocular disorders such as diabetes. No subject had previous ocular surgery or wore contact lenses. Additionally, the pupils of each subject were bigger than 3.0 mm under low mesopic conditions.

### 2.2. Procedures

Ocular aberration was measured by iTRACE (Tracey Technologies Corp., Houston, TX, USA). The experiment consisted of two sessions. In the first session, each subject underwent dark adaption in a quiet and dim room (illumination of approximately 30 lux) for at least 10 minutes. The subject was required to place his or her chin on the chin rest and use the right eye to fixate on a distant target located 4 meters away through a peekhole that was centered on the placido disk of the device and thus served as an accommodative control. The distant target was a red star sign with approximately 30 cm × 30 cm of size provided by the manufacturer, which was easy to be seen in such circumstance.

Ocular aberration of the right eye was then measured twice (measures 1-1 and 1-2). Subsequently, the subject had a rest outside the dim room with illumination of approximately 250 lux for at least 10 minutes. Then, ocular aberration was remeasured (measures 2-1 and 2-2) using the same protocol as in the first session. To avoid the potential influence of different examiners, only one examiner performed the measurements throughout the study.

### 2.3. Data Analysis

Zernike polynomials from the 2nd to 4th order were used to describe the ocular aberration. Low- and high-order total aberrations (LOA and HOA, resp.) were also summarized using the root mean square (RMS). In addition, a modulation transfer function (MTF) was employed to compare the visual quality. It is known that pupil size tends to vary between measurements and is one of the major factors that influence ocular aberration. Estimated aberration was therefore obtained by using the scaling-down technique of the instrument, with the identical pupil size determined based on the largest integer pupil size of the total four measurements.

All statistical analyses were performed using SPSS version 16.0 (SPSS 16.0 Inc., Chicago, IL, USA). The average level of ocular aberration for each session was expressed as the mean ± 1 standard deviation (1 SD), unless otherwise stated. The difference between measurements was calculated and compared within each session (intrasession) and between sessions (intersession) by using a paired *t*-test. A *p* value of less than 0.05 at two tails was considered statistically significant. To evaluate the impact of the variation between measurements, the relative difference was calculated (i.e., the difference between measurements was divided by the average of the measurements). Additionally, an intraclass correlation coefficient (ICC) was adopted to assess the intra- and intersession repeatability, as suggested by Bland and Altman [16]. An ICC > 0.75 was considered as excellent measurement reliability, ICC ≥ 0.4 was good reliability, and ICC < 0.4 was poor reliability [17].

## 3. Results

### 3.1. Variation in Pupil Size

The values of pupil sizes obtained by iTrace under natural scotopic circumstance are illustrated in Table 1. There was no statistically significant difference between the results either within sessions or between the sessions. The ICC values of the three comparisons were basically greater than 0.75, suggesting excellent intra- and intersession reliability under such circumstances.

### 3.2. Reproducibility of Ocular Aberration Measurements in Natural Scotopic Circumstances

The Zernike components measured in natural scotopic circumstances are summarized in Table 2. The intra- and intersession differences in aberration expressed by RMS, individual Zernike components, or MTF at varied spatial frequencies were generally insignificant, except for the intrasession difference of total RMS, defocus, and MTF@5cpd. However, the relative difference for the three parameters that had statistical significance was rather small (ranging from 4.31% to 7.41%), which suggested mild clinical significance. Similarly, the ICCs for all of the comparisons were generally greater than 0.4, indicating the good to excellent reproducibility of the aberration measured. It is noted, however, that the ICC for MTF@15cpd was only 0.339 in session 1 but was 0.561 in session 2 and 0.617 for both sessions.

### 3.3. Reproducibility of Ocular Aberration Estimated by Using the Pupil Scaling-Down Method

To compare ocular aberrations within the common pupil diameter, the largest integer pupil size of the total four measurements was first determined and then ocular aberration was estimated by using the pupil scaling-down method (Table 3). Similar to the data determined by using the true pupil size, the overall intra- and intersession repeatability of the results was generally good. Nevertheless, it is noted that a greater frequency of statistically significant differences and some very low ICCs (e.g., ICC of Trefoil and MTF@15cpd) was detected for estimated ocular aberrations, including total RMS, HOA total RMS, astigmatism, spherical, MTF@5cpd, MTF@15cpd, and MTF@25cpd. In addition, the relative differences of these parameters tended to be greater than those produced by using the true pupil size (e.g., 69.57% relative difference in the 2nd session for HOA total RMS, Table 3).

### 3.4. Comparison of the Measurements and Estimations of Ocular Aberrations

To further investigate the difference between the values produced from true pupil sizes and those estimated from the largest integer pupil size of all measurements, every pair of values for each aberration was compared, as shown in Table 4. As expected, due to the decrease in pupil diameter (4.36 ± 0.83 mm versus 5.15 ± 0.55 mm, *P* < 0.001), the estimated 2nd- to 4th-order Zernike coefficients were generally smaller than the corresponding measured values. Nevertheless, these differences did not reach statistical significance, except for defocus and coma. There was neither significant difference between all of the MTF values generated from estimated and the true Zernike coefficients. However, both the estimated LOA total and HOA total RMS were significantly smaller than the measured values.

## 4. Discussion

Multiple studies [12, 18, 19] have investigated the reliability of aberrometric measurements previously. Most of these studies were based on adults, and they produced controversial results. Different results could be due to different instruments but could also be due to the use of different protocols or the ages of the examined subjects. To the best of our knowledge, there are very few studies that have investigated the reliability of ocular aberration measurements in children. The present study showed that the overall intra- and intersession repeatability of ocular aberrations expressed by Zernike coefficients was satisfactory. Using a scaling-down technique with the largest integer pupil size serving as the common pupil size for different measurements could provide comparable reliability.

The influence of accommodation on ocular aberration is well documented. For instance, studies have found that the RMS of the HOAs changed sharply when the accommodation varied [12, 13]. Therefore, an unstable accommodation status would cause a declining measurement repeatability of aberration [11–13, 20, 21]. It is also well known that ocular aberration is dependent on the pupil diameter, with an increased pupil size leading to greater ocular aberration [22–24]. For the HOA profiles, the effect was found to be as high as 54.9% for observed total variability [22]. Since pupil size is influenced by both illuminance and accommodation, stable illuminance and accommodation are therefore critical prerequisites for obtaining reliable measurements of ocular aberrations. To achieve reliable measurements, in the current study, ocular aberration was measured under a scotopic condition with dark adaption in advance. Additionally, iTrace was adopted because it provided a peephole in the center of the placido disk that allowed subjects to look through the device at distant targets, which further relieved accommodation and led to reliable results. With these efforts, it was found that the pupil sizes were very well maintained between measurements both within and between sessions, leading to the overall satisfactory repeatability of the results. However, it should be pointed out that even in the current circumstance, the measurement repeatability of defocus and spherical aberrations was relatively poorer, given that these two types of aberrations are most likely to be influenced by accommodation [25, 26]. This might be also due to the fact that all participants in the present study were Chinese with deep brown iris, whose pupil sizes and the response to scotopic conditions may be significantly different from those observed in Caucasian [27, 28].

In clinical practice, it is often required to compare ocular aberrations measured with different pupil sizes at different visits. To facilitate the comparison, one approach is to acquire a common pupil size by using the scaling technique developed by Schwiegerling [29]. Pupil scaling is a mathematical procedure by which the measured Zernike coefficients for a larger pupil size are estimated for a smaller pupil size or vice versa. In the present study, it was shown that the intrasession or intersession differences of the values produced by using the scaling-down technique were not statistically significant for the majority of the Zernike components, which lends credence to using this approach in practice to compare results from different pupil sizes between visits. But how is difference between these estimated values and the truly measured values? A recent study [30] showed that the estimation of ocular aberration coefficients by either scaling down from large to smaller pupils or by scaling up from smaller to large pupils provided comparable results from clinically measured values. Although it might be difficult to directly compare the results produced by different instruments (the previous study used a Harmann-shack-principle-based aberrometer, whereas the current study used a ray-tracing-technique-based aberrometer), the comparison we found were more complicated. Specially, MTF at all spatial frequencies estimated using the scaling-down technique did demonstrate comparable results with the corresponding measured values. The majority of estimated Zernike coefficients were consistent as well with the corresponding ones, but the estimated defocus, coma, and the corresponding total aberrations were found significantly smaller than the measured values. In addition, it was observed that the variation between measurements was greater than those obtained from direct comparison between measured values. Therefore, it is suggested that comparing results by scaling the pupil down to an identical size between visits is only the second-best option, following a direct comparison with true values acquired under the circumstances in the current study. Another point worth noting is that when using the scaling technique, the rescaled magnitude should be minimal, as the present study applied the largest integer pupil size of all measurements. This is because the results from previous studies suggested that a larger scaling range tended to produce greater variability between measurements [30–32].

Caution should be taken when applying the findings of our study. First, ocular aberration is a dynamically changing parameter and each measurement actually only provided data for a static snapshot during the dynamic course [33]. To produce a reliable outcome, it is therefore critical to control the influencing factors, including pupil size and accommodation, and to adopt repeated measurements. Further, some unavoidable factors, such as the intrinsic variability of aberrations [12] and the variability associated with blinks [34], may also affect the consistency of repeated measurements. It is therefore important to acquire the aberration data at a very high speed. One advantage of the iTrace used in the study was that it can take a snapshot within 1/8th of a second [35] and thereby minimize the effects that the tear film and accommodation microfluctuations have on optical aberrations. Thus, the instrument adopted is another essential factor in determining the repeatability of ocular aberration measurements. Additionally, subjects in the current study were normal population, in whom aberrations are low to moderate. The results might be not necessarily applicable for highly aberrated eyes. Extrapolation of the current results to the populations who do not fall into the range of the myopia degree or axial length should be also avoided, because ocular aberration has been found to be related to both the refractive error [36] and axial length [37]. Therefore, further investigation in a population with wider demographic characteristics is warranted in the future.

In summary, although influencing factors such as pupil size and accommodation status impose challenges to obtaining reliable aberration measurements in children, our study showed that satisfactory intra- and intersession repeatability could be achieved under scotopic conditions by using iTrace. Scaling-down technique with the largest integer pupil size as the common pupil size offers a useful option to compare results obtained from different pupil sizes between visits, but caution should be taken because the estimated Zernike coefficients were not always comparable with the corresponding measured values.

## Figures and Tables

**Table 1:** The intrasession and intersession repeatability of the pupil sizes measured in natural scotopic circumstances.

Parameters	Process	Average	Intrasession difference	*P*	Difference/average (%)	95% LOA	ICC
Pupil size	Section 1	5.20 ± 0.53	−0.02	0.719	0.38	[−0.84, 0.80]	0.744
	Section 2	5.13 ± 0.62	−0.09	0.106	1.75	[−0.83, 0.65]	0.841
	Intersession	5.15 ± 0.55	−0.07	0.256	1.36	[−0.90, 0.76]	0.767

Note: *P*: *P* values of the intrasession difference; 95% LOA: 95% limits of agreement for the intrasession difference; ICC: intraclass correlation coefficient.

**Table 2:** The intrasession and intersession repeatability of ocular aberration measured in natural scotopic circumstances.

		Average	Intrasession difference	*P*	Difference/average (%)	95% LOA	ICC
Total RMS	Session 1	3.78 ± 1.24	−0.07	0.464	1.85	[−1.31, 1.17]	0.883
	Session 2	3.71 ± 1.20	−0.16	0.031^∗^	4.31	[−1.12, 0.80]	0.922
	Intersession	3.74 ± 1.18	−0.08	0.390	2.14	[−1.28, 1.12]	0.877

LOA total RMS	Session 1	3.73 ± 1.27	0.00	0.998	0.00	[−1.46, 1.46]	0.847
	Session 2	3.65 ± 1.23	−0.09	0.388	2.47	[−1.49, 1.31]	0.852
	Intersession	3.69 ± 1.21	−0.08	0.425	2.17	[−1.38, 1.22]	0.863

HOA total RMS	Session 1	0.33 ± 0.16	−0.01	0.654	3.03	[−0.31, 0.29]	0.668
	Session 2	0.35 ± 0.15	0.00	0.951	0.00	[−0.38, 0.38]	0.426
	Intersession	0.34 ± 0.14	0.01	0.503	2.94	[−0.23, 0.25]	0.712

Defocus	Session 1	3.64 ± 1.20	−0.07	0.644	1.92	[−2.01, 1.87]	0.719
	Session 2	3.63 ± 1.18	−0.15	0.038^∗^	4.13	[−1.09, 0.79]	0.923
	Intersession	3.64 ± 1.14	−0.01	0.941	0.27	[−1.43, 1.41]	0.824

Astigmatism	Session 1	0.59 ± 0.40	0.00	0.987	0.00	[−0.38, 0.38]	0.900
	Session 2	0.57 ± 0.35	−0.03	0.137	5.26	[−0.33, 0.27]	0.914
	Intersession	0.58 ± 0.37	−0.02	0.246	3.45	[−0.28, 0.24]	0.943

Coma	Session 1	0.24 ± 0.14	−0.02	0.403	8.33	[−0.28, 0.24]	0.682
	Session 2	0.25 ± 0.13	−0.03	0.128	12.00	[−0.31, 0.25]	0.540
	Intersession	0.24 ± 0.13	0.01	0.566	4.17	[−0.19, 0.21]	0.738

Trefoil	Session 1	0.15 ± 0.08	0.00	0.933	0.00	[−0.14, 0.14]	0.663
	Session 2	0.14 ± 0.08	0.00	0.662	0.00	[−0.12, 0.12]	0.735
	Intersession	0.14 ± 0.07	0.00	0.700	0.00	[−0.12, 0.12]	0.724

Spherical	Session 1	0.07 ± 0.07	0.01	0.295	14.29	[−0.09, 0.11]	0.728
	Session 2	0.07 ± 0.07	0.00	0.632	0.00	[−0.14, 0.14]	0.555
	Intersession	0.07 ± 0.06	0.00	0.845	0.00	[−0.10, 0.10]	0.700

Secondary	Session 1	0.06 ± 0.04	0.00	0.874	0.00	[−0.10, 0.10]	0.537
	Session 2	0.06 ± 0.04	−0.01	0.185	16.67	[−0.11, 0.09]	0.483
	Intersession	0.06 ± 0.04	0.00	0.527	0.00	[−0.08, 0.08]	0.631

MTF@5cpd	Session 1	0.55 ± 0.18	0.00	0.831	0.00	[−0.30, 0.30]	0.722
	Session 2	0.54 ± 0.18	0.04	0.033^∗^	7.41	[−0.20, 0.28]	0.811
	Intersession	0.54 ± 0.17	−0.01	0.570	1.85	[−0.19, 0.17]	0.875

MTF@10cpd	Session 1	0.28 ± 0.13	−0.01	0.358	3.57	[−0.21, 0.19]	0.723
	Session 2	0.26 ± 0.12	0.02	0.320	7.69	[−0.24, 0.28]	0.574
	Intersession	0.27 ± 0.12	−0.01	0.334	3.70	[−0.17, 0.15]	0.781

MTF@15cpd	Session 1	0.18 ± 0.09	0.00	0.894	0.00	[−0.24, 0.24]	0.339
	Session 2	0.17 ± 0.07	0.01	0.256	5.88	[−0.15, 0.17]	0.561
	Intersession	0.18 ± 0.07	−0.02	0.087	11.11	[−0.16, 0.12]	0.617

MTF@20cpd	Session 1	0.13 ± 0.06	−0.01	0.237	7.69	[−0.15, 0.13]	0.480
	Session 2	0.12 ± 0.05	0.01	0.552	8.33	[−0.09, 0.11]	0.595
	Intersession	0.12 ± 0.05	−0.01	0.102	8.33	[−0.09, 0.07]	0.764

MTF@25cpd	Session 1	0.10 ± 0.05	−0.01	0.333	10.00	[−0.11, 0.09]	0.560
	Session 2	0.10 ± 0.04	0.01	0.146	10.00	[−0.09, 0.11]	0.538
	Intersession	0.10 ± 0.04	0.00	0.443	0.00	[−0.06, 0.06]	0.734

MTF@30cpd	Session 1	0.08 ± 0.04	0.00	0.762	0.00	[−0.08, 0.08]	0.645
	Session 2	0.08 ± 0.04	0.01	0.131	12.50	[−0.07, 0.09]	0.563
	Intersession	0.08 ± 0.04;	0.00	0.324	0.00	[−0.06, 0.06]	0.701

Note: *P*: *P* values of the intrasession difference; 95% LOA: 95% limits of agreement; ICC: intraclass correlation coefficient. ^∗^Asterisk indicates statistical significance.

**Table 3:** The intrasession and intersession repeatability of the estimated ocular aberration using the pupil scaling-down method.

Parameters	Process	Average	Intrasession difference	*P*	Difference/average (%)	95% LOA	ICC
Total RMS	Session 1	2.93 ± 1.14	0.06	0.565	2.05	[−1.28, 1.40]	0.839
	Session 2	2.78 ± 1.09	−0.25	0.015^∗^	8.99	[−1.57, 1.07]	0.829
	Intersession	2.85 ± 1.07	−0.15	0.109	5.26	[−1.39, 1.09]	0.847

LOA total RMS	Session 1	2.83 ± 1.17	0.10	0.502	3.53	[−1.94, 2.14]	0.678
	Session 2	2.66 ± 1.16	−0.27	0.069	10.15	[−2.19, 1.65]	0.707
	Intersession	2.74 ± 1.11	−0.17	0.137	6.20	[−1.65, 1.31]	0.798

HOA total RMS	Session 1	0.25 ± 0.15	−0.04	0.866	16.00	[−0.32, 0.24]	0.638
	Session 2	0.23 ± 0.11	−0.16	0.010^∗^	69.57	[−0.34, 0.02]	0.739
	Intersession	0.24 ± 0.12	−0.15	0.170	62.50	[−0.31, 0.01]	0.804

Defocus	Session 1	2.78 ± 1.13	0.02	0.785	0.72	[−1.94, 1.98]	0.685
	Session 2	2.58 ± 1.14	−0.06	0.248	2.33	[−2.22, 2.10]	0.633
	Intersession	2.68 ± 1.07	−0.07	0.097	2.61	[−1.61, 1.47]	0.771

Astigmatism	Session 1	0.47 ± 0.34	0.00	0.485	0.00	[−0.36, 0.36]	0.872
	Session 2	0.43 ± 0.29	−0.04	0.029^∗^	9.30	[−0.38, 0.30]	0.849
	Intersession	0.45 ± 0.31	−0.02	0.145	4.44	[−0.34, 0.30]	0.873

Coma	Session 1	0.17 ± 0.11	0.01	0.378	5.88	[−0.17, 0.19]	0.713
	Session 2	0.17 ± 0.09	−0.02	0.128	11.76	[−0.18, 0.14]	0.723
	Intersession	0.17 ± 0.10	0.00	0.699	0.00	[−0.16, 0.16]	0.682

Trefoil	Session 1	0.11 ± 0.07	0.00	0.914	0.00	[−0.14, 0.14]	0.581
	Session 2	0.12 ± 0.10	−0.02	0.159	16.67	[−0.22, 0.18]	0.252
	Intersession	0.12 ± 0.07	0.01	0.555	8.33	[−0.19, 0.21]	0.262

Spherical	Session 1	0.06 ± 0.05	0.02	0.029^∗^	33.33	[−0.08, 0.12]	0.549
	Session 2	0.06 ± 0.05	−0.01	0.224	16.67	[−0.11, 0.09]	0.570
	Intersession	0.06 ± 0.04	0.00	0.730	0.00	[−0.08, 0.08]	0.618

Secondary	Session 1	0.05 ± 0.03	0.00	0.977	0.00	[−0.08, 0.08]	0.570
	Session 2	0.05 ± 0.03	−0.01	0.129	20.00	[−0.09, 0.07]	0.484
	Intersession	0.05 ± 0.03	0.00	0.275	0.00	[−0.06, 0.06]	0.619

MTF@5cpd	Session 1	0.60 ± 0.20	−0.01	0.478	1.67	[−0.27, 0.25]	0.810
	Session 2	0.60 ± 0.18	0.05	0.015^∗^	8.33	[−0.19, 0.29]	0.797
	Intersession	0.60 ± 0.19	0.00	0.994	0.00	[−0.14, 0.14]	0.925

MTF@10cpd	Session 1	0.33 ± 0.16	−0.03	0.071	9.09	[−0.27, 0.21]	0.743
	Session 2	0.31 ± 0.15	0.03	0.096	9.68	[−0.23, 0.29]	0.694
	Intersession	0.32 ± 0.15	−0.01	0.314	3.13	[−0.21, 0.19]	0.818

MTF@15cpd	Session 1	0.21 ± 0.11	−0.03	0.033^∗^	14.29	[−0.23, 0.17]	0.680
	Session 2	0.21 ± 0.12	0.03	0.234	14.29	[−0.33, 0.39]	0.275
	Intersession	0.21 ± 0.10	0.00	0.997	0.00	[−0.22, 0.22]	0.563

MTF@20cpd	Session 1	0.15 ± 0.08	−0.02	0.076	13.33	[−0.18, 0.14]	0.538
	Session 2	0.15 ± 0.08	0.01	0.341	6.67	[−0.15, 0.17]	0.599
	Intersession	0.15 ± 0.07	−0.01	0.460	6.67	[−0.13, 0.11]	0.698

MTF@25cpd	Session 1	0.12 ± 0.06	−0.02	0.121	16.67	[−0.14, 0.10]	0.555
	Session 2	0.11 ± 0.06	0.01	0.048	9.09	[−0.09, 0.11]	0.698
	Intersession	0.11 ± 0.06	−0.01	0.230	9.09	[−0.09, 0.07]	0.793

MTF@30cpd	Session 1	0.10 ± 0.05	−0.01	0.240	10.00	[−0.11, 0.09]	0.578
	Session 2	0.09 ± 0.05	0.01	0.063	11.11	[−0.07, 0.09]	0.675
	Intersession	0.09 ± 0.05	−0.01	0.202	11.11	[−0.07, 0.05]	0.825

Note: *P*: *P* values of the intrasession difference; 95%LOA: 95% limits of agreement; ICC: intraclass correlation coefficient. ^∗^Asterisk indicates statistical significance.

**Table 4:** Comparison of the ocular aberration between the measured and the estimated values.

Parameters	Measured value	Estimated value	Difference	*P* value	Difference/measured value (%)
Total RMS	3.74 ± 1.18	2.85 ± 1.07	−0.89	0.001^∗^	−23.80
LOA total RMS	3.69 ± 1.21	2.74 ± 1.11	−0.95	0.001^∗^	−25.75
HOA total RMS	0.34 ± 0.14	0.24 ± 0.12	−0.10	0.005^∗^	−29.41
Defocus	3.64 ± 1.14	2.68 ± 1.07	−0.96	0.000^∗^	−26.37
Astigmatism	0.58 ± 0.37	0.45 ± 0.31	−0.13	0.089	−22.41
Coma	0.24 ± 0.13	0.17 ± 0.10	−0.07	0.008^∗^	−29.17
Trefoil	0.14 ± 0.07	0.12 ± 0.07	−0.02	0.101	−14.29
Spherical	0.07 ± 0.06	0.06 ± 0.04	−0.01	0.252	−14.29
Secondary	0.06 ± 0.04	0.05 ± 0.03	−0.01	0.091	−16.67
MTF@5cpd	0.54 ± 0.17	0.60 ± 0.19	0.06	0.189	11.11
MTF@10cpd	0.27 ± 0.12	0.32 ± 0.15	0.05	0.111	18.52
MTF@15cpd	0.18 ± 0.07	0.21 ± 0.10	0.03	0.065	16.67
MTF@20cpd	0.12 ± 0.05	0.15 ± 0.07	0.03	0.067	25.00
MTF@25cpd	0.10 ± 0.04	0.11 ± 0.06	0.01	0.161	10.00
MTF@30cpd	0.08 ± 0.04	0.09 ± 0.05	0.01	0.205	12.50

Note: difference: the difference between the measured value and estimated value. *P* value: *P* value of the difference between the measured value and estimated value. ^∗^Asterisk indicates statistical significance.
